# Microbial biominers: Sequential bioleaching and biouptake of metals from electronic scraps

**DOI:** 10.1002/mbo3.1265

**Published:** 2022-02-22

**Authors:** Camino García‐Balboa, Paloma Martínez‐Alesón García, Victoria López‐Rodas, Eduardo Costas, Beatriz Baselga‐Cervera

**Affiliations:** ^1^ Animal Science (Genetics), School of Veterinary Medicine Complutense University of Madrid Madrid Spain; ^2^ Ecology, Evolution and Behavior Department University of Minnesota St. Paul Minnesota USA; ^3^ Minnesota Center for Philosophy of Science University of Minnesota Minneapolis Minnesota USA

**Keywords:** bioleaching, biouptake, electronic scraps, extremotolerant, metals, microalgae, rare earth elements

## Abstract

Electronic scraps (e‐scraps) represent an attractive raw material to mine demanded metals, as well as rare earth elements (REEs). A sequential microbial‐mediated process developed in two steps was examined to recover multiple elements. First, we made use of an acidophilic bacteria consortium, mainly composed of *Acidiphilium multivorum* and *Leptospidillum ferriphilum*, isolated from acid mine drainages. The consortium was inoculated in a dissolution of e‐scraps powder and cultured for 15 days. Forty‐five elements were analyzed in the liquid phase over time, including silver, gold, and 15 REEs. The bioleaching efficiencies of the consortium were >99% for Cu, Co, Al, and Zn, 53% for Cd, and around 10% for Cr and Li on Day 7. The second step consisted of a microalgae‐mediated uptake from e‐scraps leachate. The strains used were two acidophilic extremotolerant microalgae, *Euglena* sp. (EugVP) and *Chlamydomonas* sp. (ChlSG) strains, isolated from the same extreme environment. Up to 7.3, 4.1, 1.3, and 0.7 µg by wet biomass (WB) of Zn, Al, Cu, and Mn, respectively, were uptaken by ChlSG biomass in 12 days, presenting higher efficiency than EugVP. Concerning REEs, ChlSG biouptake 14.9, 20.3, 13.7, 8.3 ng of Gd, Pr, Ce, La per WB. Meanwhile, EugVP captured 1.1, 1.5, 1.4, and 7.5, respectively. This paper shows the potential of a microbial sequential process to revalorize e‐scraps and recover metals and REEs, harnessing extremotolerant microorganisms.

## Graphical abstract

The article shows the potential of a microbial sequential process to revalorize electronic scraps and recover metals and rare earth elements harnessing extremotolerant microorganisms.

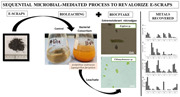

## 1 INTRODUCTION

The global volume of electronic scraps (e‐scraps) was estimated at 44.7 million metric tons in 2016, and the combined annual growth from 2017 to 2025 is calculated as a 4.1% rate (Grand View Research, 2018). The two main applications of e‐scraps are disposal (including landfilling and incineration) or recycling, which represents 12.5% of the total. E‐scraps incineration and landfilling pose a series of challenges related to space scarcity and ecological aspects. Moreover, e‐scraps represent environmental hazardous wastes due to their content of heavy metals such as lead (Pb), cadmium (Cd) or mercury (Hg), and other harmful substances (i.e., polyvinyl chloride and brominated flame retardants (Cao et al., 2016; Kiddee et al., 2013; Wäger et al., 2012; Xue et al., 2012)). This makes them a possible source of contamination of the atmosphere, sediments, and water streams. On the other hand, they contain high amounts of valuable materials, including copper (Cu), iron (Fe), gold (Au), or rare earth elements (REEs), even in higher values than natural ores (Cao et al., 2016; Cucchiella et al., 2015; Tan et al., 2015; Wibowo & Deng, 2015), making them a source of raw materials.

The change in focus of many countries toward waste‐to‐resource policies promotes mining from solid waste, instead of exploiting new sources (European Waste Electrical and Electronic Equipment Directive [2012/19/EU]). There are already examples of the harnessing of scraps, such as the regeneration of lithium electrode scraps to obtain new cathodes for lithium‐ion batteries (Zhang et al., 2016), or the recovery of copper and other precious metals from circuit boards scraps through a two steps method consisting of a combination of mechanical and electrometallurgical process (Mecucci & Scott, 2002; Veit et al., 2006), to name a few. The biotechnological approach has also been employed with this objective, and in fact, there are several publications about these applications. For example, the recovery of gold and palladium from e‐scraps with a sulfate‐reducing bacterium (Creamer et al., 2006) and biorecovery of gold from e‐scrap material through a mutated cyanogenic bacterium (Natarajan et al., 2015; Natarajan & Ting, 2014). So, there is an increasing effort to develop new technologies for the valorizing of e‐scraps. However, there are no studies that string together microbial e‐scraps leaching and elements recovery processes. One of the challenges encountered is the extreme pH values (3.6) yielded from the bioleaching process. This drawback may be overcome by employing biomass. But this option is limited by the amount of biomass used and mediated by passive accumulation (Drexler & Yeh, 2014; He & Chen, 2014). Another alternative is to employ extremotolerant microorganisms.

In this study, we investigate the bioleaching and/or accumulation abilities of different groups of microorganisms for the revalorization and metal extraction from e‐scraps. We propose a two‐phase process, the first stage of bioleaching from solid e‐scraps mediated by a bacteria consortium, followed by the second step of capture with microalgae. Specifically, for the first bioleaching phase, we made use of a bacteria consortium mainly composed of *Acidiphilium multivorum* and *Leptospirillum ferriphilum* isolated from a uranium mining site. For the second step of biocapture, we evaluated microalgae‐mediated metals recovery from leachate of e‐scraps. More specifically, *Chlamydomonas* sp. and *Euglena* sp. extremotolerant microalgae, natural inhabitants of uranium acid drainage tailings (Baselga‐Cervera et al., 2018, 2019, 2020; García‐Balboa et al., 2013). The novelty of this work is the potential synergic effect from the combination of bioleaching and bio‐uptaking to revalorize and extract valuable metals from e‐scraps.

## 2 MATERIALS AND METHODS

### 2.1 E‐scraps material

E‐scraps raw material was supplied by LYRSA‐Derichebourg, a Spanish company specializing in the recycling and management of industrial waste and e‐scraps. We employed as e‐scraps a residual powder, the fraction resulting from the recycling process after shedding and mechanical separation. E‐scraps powder was composed of solids of 1–3 mm of particle size. The characteristic materials composition of the e‐scraps is detailed in Appendix Table A1  (corresponds to “E‐scraps metallic content”).

### 2.2 Experimental microorganism isolation and culture conditions

Liquid samples of acid mine drainage were collected for the recovery of chemolithotrophic bacteria at the uranium mining sites of Saelices, and Villavieja in Salamanca (Spain) (García‐Balboa et al., 2013). Selection of a Fe‐oxidant and S‐oxidant chemolithoautotrophic bacteria consortium was performed culturing 10 ml of mine samples (Villavieja) in 90 ml of 9k medium (Silverman & Lundgren, 1959) at pH 3.05. The bacterial consortium was maintained by successive passes in a fresh 9k medium to eliminate possible traces of REE and other metals present in the original sample.

Two extremophile microalgae strains one Chlorophyceae *Chlamydomonas* sp. (ChlSG) strain (Baselga‐Cervera et al., 2018, 2019) and one Euglenaceae *Euglena* sp. (EugVP) strain (Baselga‐Cervera et al., 2020) were isolated from the uranium mine acid drainage at Saelices and Villavieja, respectively. Both strains were cultured in filtered mine water enriched with BG‐11 standard broth (Sigma‐Aldrich). Mine waters used to prepare the media were: Saelices water for ChlSG and Villavieja water for EugVP, with final pH of 3.6 ± 0.2, and 2.5 ± 0.3, respectively (mine water physicochemical characteristics can be consulted in (García‐Balboa et al., 2013). For regular maintenance, cultures were grown in 50–250 ml cell culture flasks (Greiner; Bio‐one Inc.), under continuous light conditions and at 80 µm m^−2^·s^−1^ over the waveband 400–700 nm and 22°C ± 2°C temperature. Both strains are deposited in the culture collection of the research group Albiotox, Universidad Complutense de Madrid, Spain. *Chlamydomonas sp*. ChlSG strain has been described and characterized by the research group (ChlSG strain [Baselga‐Cervera et al., 2018, 2019]).

### 2.3 Microbial consortium identification

High‐throughput sequencing was conducted at Secugen S.L. Total bacterial genomic DNA was extracted by a mechanical breakdown with zirconia beads. PCR amplification of the 16S rRNA genes was performed with the Barcoding kit (SQK‐16S024) of Nanopore, obtaining at least 2000 reads with Oxford Nanopore MinION FLO‐MIN106 technology. We obtained the different operational taxonomic units (OTUs) and compared them with the Ribosomal Database Project of Michigan State University. The. syntax file obtained was used to calculate the proportion of each OTU and the reads of the 16S were also analyzed with Epi2me of Oxford Nanopore. Low abundance OTUs (singletons, doublets, and triplets) were considered errors/artifacts and removed from the analyses (agreeing with Zhan et al., 2014).

### 2.4 Bioleaching tests and leaching ability determination

Bioleaching was carried out in 250 ml Erlenmeyer flasks with 100 ml final volume of 9k medium pH (2.05) without any additional source of energy (Fe or S) and 1% e‐scraps w/v. Two flasks were inoculated with the bacterial consortium described previously and two flasks without bacterial inoculum were used as control. Bioleaching trials were maintained in agitation (250 rpm) and at 30°C for 15 days. Bacterial inoculum consisted of 20 ml of a saturated culture of the selected bacterial consortium. During the biolixiviation experiment, the bacterial consortium thrived using e‐scraps as a primary source of energy.

Samples were collected on days 7 and 15 since the inoculum. The whole volume of each Erlenmeyer flask was sampled. Samples were filtered with a sterile filter of 0.45 microns of pore size to separate the liquid and solid phases. The liquid phase, leachate, and pellet were analyzed for 45 elements concentration and preserved for the next biouptake experiment. The solid phase was also recovered to analyze the elemental composition and compare it with the starting material.

Leaching ability (*L*_*i*_) of each element *i* was obtained as in the following equation (Savvilotidou et al., 2015):

(1) $$L_{i\;}=(C_{ie}\times V)/M,$$

where *L*_*i*_ is the leaching ability for element *i* (mg/g), *C*_*ie*_ the concentration of element *i* in the leachate at time *e* (mg/L), *M* the mass of the e‐scraps powder sample (g), and *V* the final volume of the leaching solution (L).

The extraction efficiency (*E*_*i*_) for each element was thus calculated by referring the leaching ability to the initial concentration in the e‐scraps powder, as given in the following equation (Chen et al., 2015):

(2) $$E_{i}(\%)=(L_{i}/C_{in})\times 100,$$

where *C*_*in*_ is the concentration of element *i* in the e‐scraps powder.

### 2.5 Bio‐uptake studies

Metal bio‐uptake capacity from e‐scraps leachate—recovered liquid phase—was measured in the ChlSG and EugVP strains. Cells from both strains were grown for 20 days to the stationary phase before exposure. Twelve Greiner flasks with a final volume of 10 ml were established: four with ChlSG, four with EugVP, and four without cells. Each Greiner contained 9 ml of the filtered phase from the 15 day leachate and 1 ml of the microbial culture (grown in BG‐11 culture medium) or fresh BG‐11 media in the controls. Starting pH values of the study and control dissolutions were 3.55. The initial inoculum consisted of ~3.5 ± 1.1 × 10^5^ cells for the ChlSG strain, and ~2.1 ± 0.5 × 10^3^ cells for the EugVP strain.

The whole volume of one vial of each one of the three groups (ChlSG, EugVP, and controls) was sampled at four different times: 0, 6, 144, and 288 h respectively. Each sample was processed in the following manner: first, an aliquot of the vial was used to check the appearance of the cultures and verify the viability of the cells with optical microscopy (inverted phase‐contrast fluorescence microscope [Axiovert 35; Zeiss] with a coupled camera [Axio Cam MRc; Zeiss]). Cellular densities were directly counted using a hemocytometer under the microscope (Hoshaw & Rosowski, 1973). Later, the whole volume of each culture was centrifuged (4000 rpm for 15 min), supernatants were discarded, and pellets were preserved for metal analysis.

### 2.6 Analytical methods

Multiple metal screening was conducted in the two‐phase process‐ bioleaching and bio‐uptake‐ to extract metals from e‐scraps. Metal analysis of solid samples required digestion in aqua regia solution (3 HCl: 1 HNO_3_, in volume) at 220°C that was performed in microwaves of high pressure. Analyses were performed with an Inductively Coupled Plasma Optical Emission Spectrometry (ICP‐OES; Arcos). Liquid samples were dissolved in HNO_3_ before analyses. Metal concentrations of liquid samples were performed using an Inductively Coupled Plasma Mass Spectrometry (ICP‐MS; Bruker Aurora Elite). Cellular pellets were digested in an acidic medium (0.2 ml of HNO_3_ 65% Suprapur) for 24 h at 40°C, in Teflon pumps Savillex. Later the analyses were performed with ICP‐MS.

### 2.7 Scanning electron microscopy (SEM)

For SEM, EugVP cells harvested by centrifugation were fixed in paraformaldehyde (4%)—glutaraldehyde (2.5%) for an hour at 4°C (pH was not adjusted and kept at pH ~3), postfixed samples were washed with H_2_O twice. Fixated samples were dehydrated with increasing concentrations of ethanol, dried with a critical point dryer, and gold‐coated for SEM examination.

## 3 RESULTS AND DISCUSSION

### 3.1 Metal composition of e‐scraps

E‐scraps were chemically characterized to identify the metals concentrations, presenting a complex composition. E‐scraps metallic content consisted mainly of zinc (7.3%), aluminum (5.9%), titanium (5.2), magnesium (4.6%), and important amounts of barium (3.35%), manganese (3.3%), lead (1.6%), and copper (1%), among others (a complete description of the metals analyzed can be found in Appendix Table A1). The other 75% of the scraps comprised other non‐tested metals and non‐metal content, probably plastic matter, and other organic components.

The metal composition of e‐scraps varies considerably depending on age, manufacturer, and composition, as previously reviewed by Cui and Zhang (2008). There is not a generic composition, and the content of precious metals decreases due to modern manufacturing. In contrast, the content of REE increases because of the intensive use of these elements in electric and electronic components. Different compositions may demand different microbial approaches and render different yields. For example, for printed circuit boards composition range of 10%–27% Cu, 8%–38% Fe, 2%–19% Al, 0.3%–2% Ni (Cui & Forssberg, 2007; Ilyas et al., 2007).

### 3.2 Bacterial consortia composition

As expected, the majority of the OTUs presented low abundance and were “rare” (see Supporting Information at https://doi.org/10.5281/zenodo.5819060). For the present study, we considered the role of the rare bacteria in the bioleaching process as negligible. The two major species identified in the consortium were *A. multivorum* (66% of the OTUs) an acidophilic chemoorganotrophic bacterium (Wakao et al., 1994), and *L. ferriphilum* (representing 31% of the OTUs) another acidophilic bacteria known to use the ferrous iron as electron donor (Li et al., 2020; Smith & Johnson, 2018).

*Leptospirillum* spp. is one of the most used bacteria in commercial biomining operations that require aerobic conditions and can grow with low concentrations of soluble iron (Smith & Johnson, 2018). *Acidiphilium* spp. is obligately heterotrophic, α‐Proteobacteria, that were originally described as obligate aerobes, although all classified species can reduce ferric iron to ferrous (Johnson & Bridge, 2002). This suggests that both bacteria are oxidizing and reducing iron species, creating a circle that previously has been reported with other consortia‐containing bacterial species from the same genus (Smith & Johnson, 2018). Previous studies using microbial consortia isolated from REE ore materials (Reed et al., 2016) and Kombucha (Hopfe et al., 2017, 2018) have addressed the microbial potential for recovery REE from e‐scraps powders. Their studies suggest that REE solubilization might be influenced not just by the species that compose the consortium, but also by the organic acid production of the consortia. Similar results have been also observed in fungal species (Mouna & Baral, 2019).

### 3.3 Bioleaching efficiency of the bacterial consortium

In this study, the results are presented for a 1% w/v e‐scraps concentration, using a bacterial consortium isolated from mine acid drainage and selected for iron‐oxidizing and sulfur‐oxidizing bacteria. E‐scraps presented a small particle size (0–3 mm) which increased the surface area and improved the oxidative capacity of bacteria (Ilyas et al., 2013). It is important to highlight that e‐scraps are assimilable and more complex materials than minerals. Although Fe was not directly added, it was already present.

Different leaching efficiency (*E*_*i*_) (%)—amount of the metal solubilized/initial metal concentration in the e‐scraps material—were calculated among the investigated metals after 7 and 15 days of exposure (Table 1). The consortium used, after the seventh day, presented an efficiency of more than 99% Cu, 99% Co, 99% Al, 53%, Cd, and 98% Zn, whereas Cr and Li efficiencies were below 11%. The bacterial consortium studied, leached out significant amounts of demanded metals, as well as significant amounts of other critical metals, such as or U, and even precious metals like Au (Table 1).

**Table 1:** Lixiviation and leaching ability

Element	Leachate (7 days) (ng/ml)	Leachate (15 days) (ng/ml)	Control (ng/ml)	*L*_*i*_ (7 d) (mg/g)
Ag	23 ± 5.75	<1.0	<1.0	0.002
Al	79,548 ± 19,887	46,893 ± 11,723	<1.0	7.954
As	103 ± 25.7	<10	<10	0.01
Au	23 ± 5.75	<1.0	23 ± 5.7	0.002
Ba	78 ± 19.5	36 ± 9	24 ± 6	0.007
Be	1.7 ± 0.425	1.6 ± 0.4	<1.0	>0.001
Bi	13 ± 3.25	<1.0	<1.0	0.002
Cd	145 ± 36.2	85 ± 21.25	1 ± 0.25	0.015
Ce	82 ± 20.5	52 ± 13	<1.0	0.008
Co	1234 ± 308	356 ± 89	3.1 ± 0.775	0.123
Cr	756 ± 189	13 ± 3.25	6 ± 1.5	0.076
Cu	11,030 ± 2757	7337 ± 1834	206 ± 51.5	1.103
Dy	7.1 ± 1.77	5.8 ± 1.45	<0.10	>0.001
Er	2.4 ± 0.6	1.9 ± 0.475	<0.10	>0.001
Eu	3 ± 0.75	2.4 ± 0.60	<0.10	>0.001
Fe	90,000 ± 22,500	365 ± 91.2	<10	9
Gd	14 ± 3.5	6.4 ± 1.60	<0.10	>0.001
Ho	1.1 ± 0.275	0.75 ± 0.185	<0.10	>0.001
La	52 ± 13	34 ± 8.5	<0.10	0.005
Li	120 ± 30	87 ± 21.75	68 ± 17	0.012
Lu	0.46 ± 0.155	0.29 ± 0.07	<0.10	>0.001
Mn	7414 ± 1853	6476 ± 1619	<10	0.741
Mo	137 ± 34.2	<1.0	33 ± 8.25	0.014
Nb	3.9 ± 0.975	<1.0	<1.0	>0.001
Nd	103 ± 25.7	63 ± 15.7	<0.10	0.01
Ni	2472 ± 618	1757 ± 439	28 ± 7	0.247
Pb	5688 ± 1422	330 ± 82.5	<1.0	0.569
Pr	25 ± 6.25	14 ± 3.5	<0.10	0.003
Pt	<1.0	<1.0	<1.0	>0.001
Rb	21 ± 5.25	20 ± 5	18 ± 4.5	0.002
Sb	11 ± 2.75	<1.0	7.5 ± 1.87	0.001
Sm	6.6 ± 1.65	5.4 ± 1.35	<0.10	>0.001
Sn	102 ± 25.5	<10	<10	0.01
Sr	718 ± 179.5	637 ± 159	121 ± 30.2	0.072
Tb	1 ± 0.25	1 ± 0.25	<0.10	>0.001
Th	8.8 ± 2.2	2 ± 0.5	<1.0	>0.001
Ti	769 ± 192	<1.0	<1.0	0.077
Tm	0.38 ± 0.095	0.28 ± 0.07	<0.10	>0.001
U	253 ± 63.25	158 ± 39.5	<1.0	0.025
V	216 ± 54	<1.0	2 4 ± 6	0.022
W	16 ± 4	<1.0	3.3 ± 0.82	0.002
Y	50 ± 12.5	40 ± 10	<0.10	0.005
Yb	2.3 ± 0.575	1.7 ± 0.4	<0.10	>0.001
Zn	71,518 ± 17,879	68,387 ± 17,096	23 ± 5.7	7.152
Zr	157 ± 39.2	<1.0	<1.0	0.016

*Note*: Values (value ± analytical error) represent the metals lixiviated in the leachates (at 7 and 15 days) and control from e‐scraps mediated by a bacterial consortium. Leaching ability was presented as L_i_ on day 7.

The main economic driver of bioleaching is the recovery of precious metals, such as Pd, Ag, or Au, and critical metals like Cu, Al, Ni, Zn, Li, or U and REEs. Many researchers have investigated the processes of bioleaching of metals from e‐scraps (as previously reviewed by Ilyas & Lee, 2014, and Gu et al., 2016). Leaching efficiency depends on the source (Brandl et al., 2001; Qu & Lian, 2013; Tran et al., 2011), microorganisms (Brandl et al., 2001; Ilyas et al., 2013; Vestola et al., 2010), and leaching conditions. Metal bioleaching from e‐scraps is complex due to the relation between the above‐mentioned factors that affect the efficiency of the process.

Solid/liquid ratio plays a crucial role in the effectiveness of the process; high pulp densities impact the metal solubilization through arresting cellular growth (Madrigal‐Arias et al., 2015; Mishra et al., 2008). As in our case, an intermediate pulp density, 1% w/v, was selected to reduce the negative effect of high densities (Shaikh Shafikh et al., 2018; Tipre & Dave, 2004), according to previous studies that considered 1% (w/v) the optimum operation condition to extract metal (Marra et al., 2018). Higher e‐scraps concentrations (5%–20% w/v) have been assessed to increase the yield, however, they also observed toxic effects on non‐adapted cultures and unwashed e‐scraps (Natarajan & Ramanathan, 2015; Shaikh Shafikh et al., 2018). Shorter leaching time and better efficiency have been obtained in studies with adapted consortiums and low pulp density. For example, Ilyas et al. (2007) compared wild cultures, adapted cultures, and an adapted consortium of thermophilic bacteria, at washed and unwashed e‐scraps at 10 g/l in 18 days. The adapted consortium showed a better leaching performance (more than 89% Cu, 81% Ni, 79% Al, and 83% Zn). Wu et al. (2018) also investigated a free‐living bacterial consortium, obtaining 100% of Cu leaching efficiency in 2 h with a 5 g/l pulp. Our free‐living consortium presented better leaching kinetics and overall higher efficiency for demanded metals, Al, Cu, or Zn, than Ilyas et al. (2007), and the same efficiency but worse kinetics for Cu when compared with Wu et al. (2018) results.

Selective mobilization of distinct metals is mediated by oxidation/reduction reactions or by proton attraction‐forming acids. Therefore, metals with lower standard electrode potential, such as Al and Zn, are oxidized and dissolved preferentially to other metals that require longer times such as Cu. Similar trends were observed in the present work and previously reported by other authors (Marra et al., 2018; Utimura et al., 2017). Metal dissolution is also influenced by its solubility. In the case of precious metals, acidophiles are widely used for gold mining operations, but normally copper is dissolved, and the precious metal reminds concentrated in the solid phase, being extracted by subsequent processes (Brandl et al., 2001; Das et al., 2017). In this study, we observed the leaching of small amounts of Au on day 7, in contrast with other acidophilic bacteria bioleaching studies (Marra et al., 2018). Gold mobilization from e‐scraps and waste electrical and electronic equipment (WEEE) has been mainly performed via complexolysis, a mechanism mediated by heterotrophic cyanogenic microorganisms (Arshadi et al., 2016; Kumar et al., 2018; Pradhan & Kumar, 2012).

Varied amounts of REEs were also transferred from e‐scraps to the dissolution phase: Ce, Dy, Er, Eu, Gd, Ho, La, Lu, Nd, Pr, Sm, Tb, Tm, and Yb (Table 1). REEs were not found in the control solution, indicating that REE mobilization was mediated by the consortium activity. REE microbial mobilization has mainly focused on the extraction from native minerals (Desouky et al., 2016; Işıldar et al., 2019; Shin et al., 2015). Moreover, the mechanisms of chemical and biochemical interaction among microorganisms with REEs are still not well‐understood (Barmettler et al., 2016), but it can be assumed that general mechanisms of acidolysis and redoxolysis are involved. Ambaye et al. (2020) revised the current literature of REEs recovered from industrial and electronic waste. Among the revised studies, only a couple studied REEs bioleaching from WEEE and e‐scraps. Beolchini et al. (2012) focused on the recovery of metals, including yttrium (Y) with a 70% yield from fluorescent powders, utilizing a mixture of Fe/S oxidizing bacteria. Marra et al. (2018) performed a two‐steps bioleaching process from e‐waste shedding dust, first extracting REEs such Ce, Eu and Nd, Y, and La (mobilizing up to 99% for the three first days, and up to 80% for Y and La, after 8 days) by acidophilic bacteria. In a second step with cyanide‐producing bacteria, Marra et al. (2018) observed no REEs extraction suggesting that complexolysis is not involved in REEs mobilization. In our study Ce, La, Y, and Nd reached values between 5 and 10 μg/g on day 7.

Future studies with different pulp densities, adaptation to e‐scraps, and varying conditions are required to assess the real potential of this consortium. However, the bioleaching profile of the isolated consortium showed the feasibility to solubilize some metals and REEs with high efficiency and below the average time intervals.

### 3.4 Leachate solid precipitation

Analysis on day 15 showed a decrease in the concentration of dissolved metals in the leachate composition and an increase in the metal amount of the solid phase (Tables 1 and A1). These results reflect a chemical reaction of the formation of yellowish‐brown precipitates, compatible with jarosites. Jarosites are basic ferric hydroxy sulfate minerals with the chemical formula of MFe_3_(SO4)_2_(OH)_6_. These minerals are common precipitates in the conditions of these types of reactions (Daoud & Karamanev, 2006). Ferric hydroxy sulfate minerals can form reactive surfaces and diffusion barriers, slowing down the flux from reactants to products (Nemati et al., 1998; Vestola et al., 2010). Thus, jarosite formation could be used as a signal to establish the optimal moment to stop the reaction and recover the enriched solution.

The metallic composition of the solid phase was also analyzed, before the jarosite formation at day 7, and the end of the experiment at day 15 (Table A2). The percentage of metals solubilized was more than 50% Cu, 55% Ni, 34% Al, 44% Li, and 94% Zn. The solid phase after the bioleaching step consisted of the remaining e‐scraps, consortium cells, and precipitates. Our interest in analyzing the composition of the solid phase after bioleaching is related to its disposal. The final disposal of the residue, either landfilling or energetic valorization at a cement plant, is related to its composition. Cement plant admission requirements are highly influenced by the metal content of the solid deposits (Joseph et al., 2018). It is evident from these data that the bacterial consortium oxidation activity reduced the concentration of metals of the solid phase of all the main elements analyzed. It is particularly remarkable the significant decline in the case of Al, Ba, Mg, Ti, and Zn after 7 days. An extra benefit is not obtained if cultures are maintained longer because of the reactions of precipitation, as described just above. From day 7 to day 15, due to the precipitation reactions described above, the solid phase is enriched in metals such as Al, Cu, Ni, or Zn, and further depleted in Cd, Mn, P, and Ti.

### 3.5 Microalgae metals and REEs removal from leachate media

**Figure 1:**
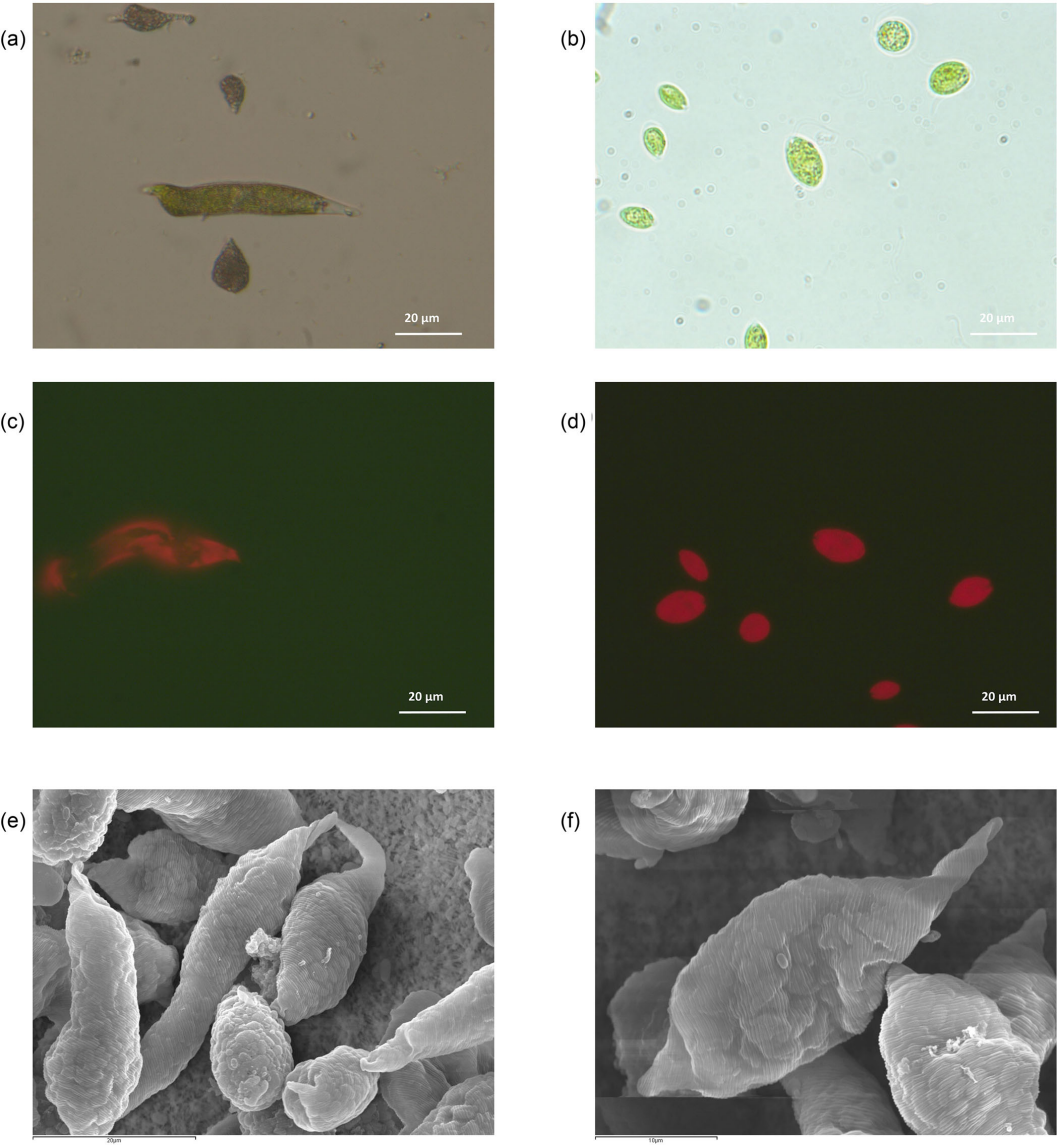
Microscope images and autofluorescence micrographs of EugVP (a and c) and ChlSG (b and d), isolated from an acid mine drainage. (e and f) SEM micrographs of EugVP. SEM, scanning electron microscopy

In this context, we have capitalized on the advantages of two extremotolerant microalgae, ChlSG and EugVP strains (Figure 1), that evolved tolerance to acid mine drainages and have been thriving in these extreme anthropogenic environments ever since. Both strains are tolerant of high metals concentrations and acid pH values below 3.5, making them tailored to the leachate conditions. We exposed both strains to a 15 day leachate solution, over 12 days, to address the removal performance over time of these two extremotolerant microalgae. Differences between the leachate at Day 15 and the removal leachate (leachate at Day 15 enriched with BG‐11 broth), like increased Fe or the presence of Mo, can be explained by the microalgae broth addition.

**Figure 2:**
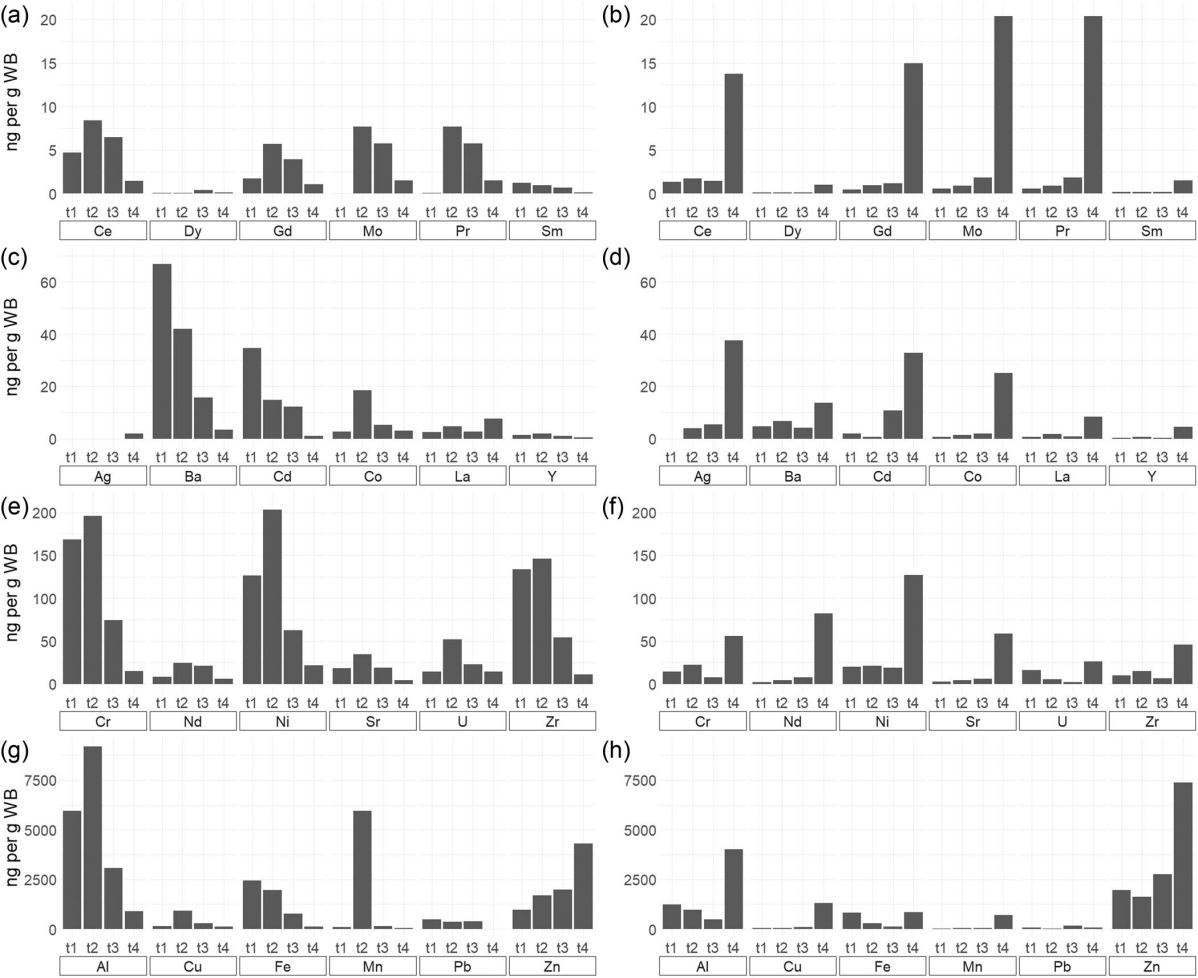
Concentration profile (ng) of metals uptake by microalgae pellets. (a), (c), (e), and (g) The amounts of each metal recovered by EugVP over time (*t*_1_, *t*_2_, *t*_3_, and *t*_4_, correspond to 0 h, 8 h, 114 h, and 288 h, respectively). (b), (d), (f), and (h) The amounts recovered by ChlSG

Over the removal experiments, both strains performed well under the new environment. Viability and growth did not appear to be impaired by the composition of the leachate (3.6 pH‐value and more than 30 elements dissolved in the solution which concentration ranged from 1 to 1000 ng/ml). Bio‐capture occurred in 24 out of 33 analyzed elements present in the control removal dissolution for both strains. From the element scrutiny in the liquors, the removal was very satisfactory for many elements. In general terms, both species retained up to µg of Al, Fe, Zn, Pb, Cu, and Mn, and considerable amounts of other metals, with the better overall performance of ChlSG in terms of ng per g of wet biomass (WB) pellets (Figure 2). EugVP presented a better performance over time (Figure 2). REEs uptake reached significant concentrations in the cellular pellets. GhlSP uptake larger REEs amounts, up to 14.97 ng Gd, 20.36 ng of Pr, 13.77 ng of Ce, or 8.38 ng of La per g of WB.

Neither of the strain pellet chemical analyses showed detectable concentrations of titanium, beryllium, or these REEs: Eu, Ho, Lu, Tb, TM, and Yb, elements that were present in the removal liquors. In the case of Erbium, chemical analyses were inconsistent, probably an artifact of the sensitivity of the analysis. Control liquor metals concentrations remained constant along with the trials. There was no chemical precipitation mediated by non‐biological factors in the experimental conditions (detailed element analyses can be found in Table A2).

It is not surprising to observe differences between the strains given that both strains, despite being single cell flagellated eukaryotes grouped as microalgae, are completely different organisms that have structural and functional differences (Figure 1). In terms of structure, ChlSG is microscopic unicellular oval shape biflagellate green microalgae approximately 10 µm in diameter with a multilayer cell wall. EugVP is microscopic elongated and spindle‐shaped single‐celled protozoans of approx. 40–60 µm long and 8–12 µm wide with two unequal length flagella and covered by a composite membrane called pellicle devoid of cellulose. ChlSG presents both sexual and asexual reproduction, whereas EugVP only reproduces by binary fission. EugVP, unlike ChlSG, can also nourish heterotrophically. Furthermore, both strains showed notable differences in growth performances. After 12 days EugVP appears to remain exponential phase reaching concentrations of ~2.8 × 10^5^ cells per ml and ChlSG is probably in late stationary phase with ~4.8 × 10^6^ cells per ml showing a drop in the cell densities compared with day 6 (Table A3).

To compare across the strain efficiency over time, we also estimated the amount of element uptake by cell, and by estimated cell volume, as possible indicators of the capacity to remove metals from the solution (detailed description of the calculations is described in Supporting Information at https://doi.org/10.5281/zenodo.5819060). Although the amount uptaken per cell is better in EugVP, ChlSG uptake larger amounts per biovolume. Taking this into account, and the higher growth rate of ChlSG, we can infer that ChlSG presented better removal efficiency. *Chlamydomonas* spp. cellular wall displays a strong affinity for metallic cations (Collard & Matagne, 1990), it lacks cellulose but consists of a multilayer of hydroxyproline‐rich glycoproteins. The plentiful group's anionic carboxyl of pectin and glycoproteins play a dominant role in binding metal ions, making stronger bonds to metal ions that have lost the hydration sphere (Van Custem & Gillet, 1982). Therefore, *Chlamydomonas* spp. has been widely used in metal biosorption studies from different kinds of liquors (Baselga‐Cervera et al., 2018; Bayramoǧlu et al., 2006; Flouty & Estephane, 2012; Wan Maznah et al., 2012). *Euglena* spp. pellicle consists of a complex proteinaceous layer underlain by microtubules, with a high concentration of charged and polar amino‐acid and sugar residues (Nakano et al., 1987). However, those polar components are very likely in higher proportion in strains cultured anaerobically (Santiago‐Martínez et al., 2015). Regarding previous studies of biosorption of metals with species of *Euglena* genus, Santiago‐Martínez et al. (2015) observed lower or negligible Cd biosorption at 100 µM concentrations. Moreno‐Sánchez et al. (2017) suggest that this removal mechanism is unproductive, suggesting the intracellular accumulation as the efficient research direction. In terms of intracellular accumulation, studies with cadmium showed 33 times higher accumulation of *E. gracili*s, in comparison with two *Chlamydomonas* spp. (Moreno‐Sánchez et al., 2017), and high zinc accumulation (Sánchez‐Thomas et al., 2016). Studies in *Chlamydomonas* spp. found 1.8 and 2.75 more biosorption than bioaccumulation of Cu and Pb respectively (Flouty & Estephane, 2012; Flouty & Khalaf, 2015). Although it was not the purpose of this study, if one process is prevalent in either of the species, observed differences could be related to the uptake process.

The uptake kinetics observed varied depending on the strain and element. There were cases where the concentration of an element was constant during the 288 h of the experiments. This was the case of Ba in both strains or Fe, Cd, and Sm in the case of EugVP. In contrast, there are examples where the bio‐uptake kinetics increased over time. For example, the precious metal Ag or another as Co, Cu, Mn, Mo, and Zn in both strains. More detailed trials focused on kinetic aspects would be relevant to elucidate this aspect. The phenomenon of desorption was also observed with some elements: U (ChlSG), Fe, and Pb in both strains. The fact that EugVP was at its exponential phase while ChlSG reached the stationary phase might explain the possible desorption observed at 12 days in ChlSG. Other factors, like initial metal concentration, elements' competition for the bidding sites (Aharchaou et al., 2020), or acquired adaptive mechanisms, might influence the observed kinetics.

There is ample literature on research and efficiency of biological removal of metals from leachates and metals solutions (as reviewed by Carvajal‐Flórez & Cardona‐Gallo, 2019; Dobson & Burgess, 2007; He & Chen, 2014; Ismail et al., 2014; Johnson & Hallberg, 2005, just to name a few). Işıldar et al. (2019) reviewed the literature on biosorption of REEs and other metals of interest from WEEE leachates. For instance, on microalgae death biomass, Birungi and Chirwa (2014) studied the biosorption of La by *C. reinhardtii* (142.86 mg/g) and *Chlorella vulgaris* (74.6 mg/g) and other four species of microalgae. Kucuker et al. (2017) *C. vulgaris* addressed Nd (157.21 mg/g) and Wojciech Heilmann et al. (2015) addressed 17 species of microalgae being the more efficient *Tetraselmis chuii* and *Calothrix brevissima* in Nd bioaccumulation (efficiency of 51.92 and 69.23 mg/g, respectively). The present study is addressing biouptake from complex leachate liquor with living microalgae, solving the pH adaptation mentioned as a bottleneck needed before biosorption (Işıldar et al., 2019). The use of alive biomass also implies enzymatic processes that, although further studies should be conducted, could involve valuable mechanisms for unresolved problems like specific captures toward some metals. We have capitalized on extremophilic microalgae already adapted to acid conditions and dissolved metals. Further studies are needed to elucidate the mechanisms and efficiencies involved in the removal of metals with living microalgae from complex leachate liquors.

## 4 CONCLUSIONS

We harvested e‐scraps using a sequential microbial‐mediated process developed in two stages: bioleaching using an acidophilic ferrous iron‐oxidizing bacteria consortium and the biouptake of metals from the leachate by extremophilic microalgae strains. Results showed that this biotechnological methodology can be used to recover metals from e‐scraps. Recovery values of the bioleaching stage were nearly 100% for Cu, Co, Al, and Zn. ChlSG pellets accumulated µg of Zn, Al, Cu, and Mn, and ng of REEs. This research represents an approach to the complete valorization of e‐scraps with extremotolerant microorganisms.

## CONFLICT OF INTERESTS

None declared.

## ETHICS STATEMENT

None required.

## AUTHOR CONTRIBUTIONS

**Camino García‐Balboa**: conceptualization (lead), data curation (equal), formal analysis (equal), investigation (equal), writing–original draft (supporting), writing–review and editing (equal). **Paloma Martínez‐Alesón**: conceptualization (supporting), formal analysis (supporting), investigation (equal), writing–original draft (supporting), writing–review and editing (equal). **Victoria López‐Rodas**: funding acquisition (lead), supervision (equal), writing–review and editing (equal). **Eduardo Costas**: funding acquisition (lead), supervision (equal), writing–review and editing (equal). **Beatriz Baselga‐Cervera**: conceptualization (supporting), data curation (equal), formal analysis (equal), funding acquisition (supporting), visualization (lead), writing–original draft (lead), writing–review and editing (lead).

## Data Availability

All data generated during the current study are provided in full in this paper apart from the data matrix for all the OTU reads from the consortium and metals biouptake normalized by microalgae biomass, which is available in the Zenodo repository at https://doi.org/10.5281/zenodo.5819060.

## APPENDIX A

**Table A1:** Chemical analyses of metal content

Parameter (µg/g)	E‐scraps metallic content (*t*_0_)	Solid phase (*t*_7_)	Solid‐phase (*t*_15_)	*E*_*i*_ (%) (*t*_7_)	*E*_*i*_ (%) (*t*_15_)	Metal solubilized (%) (*t*_7_)	Metal solubilized (%) (*t*_15_)
Al	5862 ± 1465	3823 ± 955	4038 ± 1009	>99	80.0	34.8	31.1
Ba	3347 ± 836	2175 ± 543	2175 ± 543	0.2	0.1	35.0	35.0
Ca	6 ± 1.5	1.5 ± 0.3	3.8 ± 1	n/a	n/a	75.0	36.7
Cd	27 ± 6.7	9 ± 2.2	1.2 ± 0.3	53.7	31.5	66.7	95.6
Co	92 ± 23	36 ± 9	39 ± 9	>99	38.7	60.9	57.6
Cr	694 ± 173	524 ± 131	533 ± 133	10.9	0.2	24.5	23.2
Cu	1007 ± 251	470 ± 117	571 ± 142	>99	72.9	53.3	43.3
Li	142 ± 35	79 ± 19	79 ± 19	8.5	6.1	44.4	44.4
Mg	4587 ± 1146	2179 ± 544	2779 ± 694	n/a	n/a	52.5	39.4
Mn	3298 ± 824	2559 ± 639	2265 ± 566	22.5	19.6	22.4	31.3
Mo	98 ± 24	79 ± 19	81 ± 20	14	0	19.4	17.3
Ni	430 ± 107	186 ± 46	232 ± 58	57.5	40	56.7	46.0
P	776 ± 194	338 ± 84	212 ± 53	n/a	n/a	56.4	72.7
Pb	1623 ± 405	891 ± 222	918 ± 229	35	2	45.1	43.4
Ti	5179 ± 1294	3412 ± 853	2492 ± 623	1.5	0	34.1	51.9
Zn	7279 ± 1819	402 ± 100	640 ± 160	>98	93.8	94.5	91.2

*Note*: The content of the main metals (value ± analytical error) analyzed in e‐scraps raw material, solid‐phase at the initial time (*t*_0_), after bioleaching for 7 (*t*_7_) and 15 (*t*_15_) days, the leaching efficiencies *E*_*i*_ (%) (Equation 2), and the percent of metal solubilized estimated from e‐scraps and solid‐phase metallic content change are shown.

Abbreviation: n/a, not applicable.

**Table A2:** Biouptake experiment metals content (ng/ml) in the control leachate over time (value ± analytical error)

Element	0 h	8 h	144 h	288 h
Ag	2.6 ± 0.65	3.6 ± 0.9	1.4 ± 0.35	1 ± 0.25
Al	28,954 ± 7238	29,220 ± 7305	34,257 ± 8564	40,460 ± 10,115
Ba	19 ± 4.75	20 ± 0.5	20 ± 5	23 ± 5.7
Be	0.8 ± 0.2	1.2 ± 0.3	1.5 ± 0.37	2.1 ± 0.52
Cd	75 ± 18.7	73 ± 18.25	80 ± 20	86 ± 21.5
Ce	68 ± 17	66 ± 16.5	71 ± 17	78 ± 19.5
Co	266 ± 66	262 ± 65.5	298 ± 74	343 ± 85.75
Cr	13 ± 3	20 ± 5	13 ± 3	16 ± 4
Cu	8154 ± 2038	8134 ± 2033	9121 ± 2280	10,324 ± 2581
Dy	7.3 ± 1.8	7.1 ± 1.77	7.9 ± 1.9	8.1 ± 2.02
Er	1.8 ± 0.45	2 ± 0.5	2.1 ± 0.52	2 ± 0.5
Eu	2 ± 0.5	2 ± 0.5	2.2 ± 0.5	2.3 ± 0.57
Fe*	839 ± 209	1490 ± 372	988 ± 247	1038 ± 259
Gd	120 ± 30	117 ± 29	128 ± 32	137 ± 34
Ho	2.1 ± 0.5	2 ± 0.5	2.3 ± 0.57	2.5 ± 0.62
La	35 ± 8.7	34 ± 8.5	37 ± 9.2	40 ± 10
Lu	0.25 ± 0.06	0.24 ± 0.06	0.27 ± 0.06	0.3 ± 0.075
Mn	6466 ± 1616	6497 ± 1624	7430 ± 1857	8499 ± 2124
Mo	13 ± 3.2	13 ± 3.2	13 ± 3.2	14 ± 3.5
Nd	572 ± 143	555 ± 138	613 ± 153	672 ± 168
Ni	1404 ± 351	1389 ± 347	1582 ± 395	1805 ± 451
Pb	26 ± 6.5	27 ± 6.7	30 ± 7.5	33 ± 8.2
Pr	150 ± 37	144 ± 36	161 ± 40	175 ± 43.75
Sm	4.8 ± 1.2	5.2 ± 1.3	5.1 ± 1.2	5.4 ± 1.3
Sr	447 ± 111	456 ± 114	500 ± 125	553 ± 138
Tb	1.1 ± 0.2	1.1 ± 0.27	1.2 ± 0.3	1.3 ± 0.32
Ti	1.2 ± 0.3	2.3 ± 0.57	2 ± 0.5	1.9 ± 0.47
Tm	0.23 ± 0.05	0.21 ± 0.05	0.22 ± 0.55	0.27 ± 0.06
U	85 ± 21	85 ± 21	93 ± 23	102 ± 25
Y	33 ± 8.2	33 ± 8.2	36 ± 9	41 ± 10
Yb	1.7 ± 0.42	1.5 ± 0.37	1.7 ± 0.42	2 ± 0.5
Zn	53,818 ± 13,454	53,147 ± 13,286	58,805 ± 14,701	66,746 ± 16,686
Zr	1.3 ± 0.32	1.8 ± 0.45	2.1 ± 0.5	2.8 ± 0.7

*Calculated as ng per kg.

**Table A3:** Cell densities of both microalgae strains counted in a hemocytometer

	ChlSG strain	EugVP
Initial inoculum (cells)	3,576,000	21,200
8 h (cells/ml)	3.57 ± 1.12 × 10^5^	21.2 ± 4.98 × 10^2^
114 h (cells/ml)	5.24 ± 1.45 × 10^6^	51.8 ± 18.87 × 10^3^
228 h (cells/ml)	4.77 ± 1.26 × 10^6^	2.78 ± 1.13 × 10^5^
